# PLK2 modulation of enriched TAp73 affects osteogenic differentiation and prognosis in human osteosarcoma

**DOI:** 10.1002/cam4.3066

**Published:** 2020-04-29

**Authors:** Wenhu Li, Xianliao Zhang, Xinhua Xi, Yufa Li, Hong Quan, Shifeng Liu, Liqi Wu, Penghuan Wu, Wenxing Lan, Yongjun Shao, Haomiao Li, Kebing Chen, Zhengbo Hu

**Affiliations:** ^1^ Department of Orthopedics Shaoguan First People's Hospital Affiliated to Southern Medical University Shaoguan China; ^2^ Orthopedics Center Zhujiang Hospital of Southern Medical University Guangzhou China; ^3^ Department of Orthopaedics The Affiliated Yuebei People's Hospital of Shantou University Medical College Shaoguan China; ^4^ The Second School of Clinical Medicine Southern Medical University Guangzhou China; ^5^ Department of Pathology Guangdong provincial people's Hospital & Guangdong, Academy of Medical Sciences Guangzhou China; ^6^ Orthopedics Center Dongguan Eighth People's Hospital Dongguan China; ^7^ Orthopedics Center The Third Affiliated Hospital of Southern Medical University Orthopedics institute of Guangdong Province Guangzhou China

**Keywords:** osteogenic differentiation, Osteosarcoma, PDX, PLK2, TAp73

## Abstract

There are three subtypes of undifferentiated human conventional osteosarcoma (HCOS): osteoblastic osteosarcoma (OOS), chondroblastic osteosarcoma (COS), and fibroblastic osteosarcoma (FOS). HCOS also exhibits heterogeneous pathological maldifferentiation in individual patients. Currently, the mechanism regulating HCOS differentiation remains unclear, and therapies are ineffective. Osteopontin (OPN) and osteocalcin (OCN) are markers of osteoblast maturation, and their expression is inhibited in HCOS. A previous study found that PLK2 inhibited TAp73 phosphorylation and consequent anti‐OS function of TAp73 in OS cells with enriched TAp73. TAp73 was also reported to regulate bone cell calcification. Here, OOS was found to have higher TAp73 levels and PLK2 expression than those in COS, which is correlated with HCOS maldifferentiation according to Spearman analysis and affects patient prognosis according to Kaplan‐Meier survival analysis. In the conventional OS cell‐line Saos2 and in patient‐derived xenograft OS (PDX‐OS) cells, increased PLK2 expression owing to abundant TAp73 levels affected OPN and OCN content as measured by RT‐PCR and Western blotting, and alizarin red staining showed that PLK2 affected calcium deposition in OS cells. In addition, PLK2 inhibition in PDX‐OS cells prohibited clone formation, as indicated by a clonogenic assay, and sensitized OS cells to cisplatin (CDDP) (which consequently limited proliferation), as shown by the CCK‐8 assay. In an established PDX animal model with abundant TAp73 levels, PLK2 inhibition or CDDP treatment prevented tumor growth and prolonged median survival. The combined therapeutic effect of PLK2 inhibition with CDDP treatment was better than that of either monotherapy. These results indicate that increased PLK2 levels due to enriched TAp73 affect osteogenic differentiation and maturation and OS prognosis. In conclusion, PLK2 is a potential target for differentiation therapy of OS with enriched TAp73.

## Graphical abstract

This study indicates that PLK2 modulation of highly abundant TAp73 to regulate the clonogenicity, proliferation, osteogenic differentiation, and maturation of OS cells. In addition, PLK2 is a potential therapeutic target in preclinical PDX hosts that can sensitize tumors to the antitumor effect of CDDP and prolong the medium survival of the hosts. In conclusion, PLK2 may be an ideal target for OS differentiation therapy.

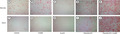

## 1 INTRODUCTION

Osteosarcoma (OS) is the most common malignant bone tumor in adolescents. The combination regimen comprising methotrexate, cisplatin, and doxorubicin (MAP) is the current standard for neoadjuvant chemotherapy.^1^, ^2^ Neoadjuvant chemotherapy combined with surgery significantly improves the 5‐year survival rate of patients, but this rate has remained stagnant over the last 30 years.^3^, ^4^, ^5^, ^6^, ^7^, ^8^ In addition, factors such as chemical toxicity, drug resistance, genetic heterogeneity of the tumor, pathological diversity, and tumor cell maldifferentiation seriously affect clinical treatment and patient prognosis.^9^, ^10^ Therefore, exploration of new OS treatments is urgently needed.^11^, ^12^

The more differentiated the tumor cells are, the lower the degree of malignancy, and the better the therapeutic effect. Although the incidence of OS is relatively low, OS pathological classification is highly complex.^13^ Only human conventional osteosarcoma (HCOS) can be categorized into three pathological subtypes of poorly differentiated cancers,^14^, ^15^ namely, osteoblastic osteosarcoma (OOS), chondroblastic osteosarcoma (COS), and fibroblastic osteosarcoma (FOS).^16^, ^17^ Even within the same patient, HCOS tissues are heterogeneous with different degrees of cell differentiation.^18^ Therefore, inducing OS differentiation and maturation is also a significant target for treating OS.^18^, ^19^

The concept of differentiation therapy is derived from the fact that hormones or cytokines may promote differentiation in vitro, thus irreversibly changing the cancer cell phenotype.^20^ The landmark success of differentiation therapy is the high cure rate for acute promyelocytic leukemia with the combination of retinoic acid and arsenic.^21^, ^22^ Recently, drugs that induce differentiation in a variety of primary tumor cells,^23^, ^24^, ^25^ including OS cells,^26^, ^27^, ^28^, ^29^ have been identified, which indicates that differentiation therapy has potential clinical application.

The heterogeneity of OS pathological differentiation is closely related to tumor gene mutations.^30^, ^31^, ^32^ The mutation rate of the endogenous antitumor gene p53 in OS is over 70%.^33^ Fortunately, the p53 family member p73 gene product TAp73 can partially replace p53 to perform antitumor functions and exhibits almost no mutations.^34^, ^35^, ^36^ It has been reported that TAp73 has the ability to regulate the differentiation of tumor cells and affects the survival and prognosis of patients.^37^ Animal studies have suggested that p73 plays an important role in bone development and, that once p73 deficiency is established, survival is diminished.^38^, ^39^, ^40^, ^41^ Additionally, high expression of TAp73 has been observed in some tumors, but its antitumor function was lost.^42^, ^43^, ^44^

PLK2 is a cell‐cycle regulatory gene that affects tumor cell‐cycle progression, cell proliferation, and individual bone development.^45^ A previous study ^46^ found that in a conventional OS cell line, PLK2 inhibited TAp73 phosphorylation and TAp73 anti‐OS function in the context of abundant TAp73. Inhibiting PLK2 enhanced the sensitivity of OS cells to cisplatin (CDDP). Several studies have shown that TAp73 can regulate osteogenic differentiation ^26^, ^47^, ^48^, ^49^, ^50^ and increase the expression of osteogenic differentiation markers such as osteopontin (OPN) and osteocalcin (OCN) ^51^, ^52^ in conventional OS cell lines lacking p53, such as Saos2 and MG63 cells, but whether TAp73 affects the expression of chondrogenic differentiation markers such as Sox9 and Collagen2 ^53^, ^54^ is still unknown. However, it remains unclear whether PLK2 affects OS osteogenic differentiation and maturation through its interaction with TAp73 and has an effect on the prognosis of patients.

Due to OS heterogeneity in tumor genes and pathological maldifferentiation,^14^, ^15^, ^18^, ^19^, ^30^ research results for one pathological type of OS are difficult to replicate in other types.^31^ The patient‐derived xenograft (PDX) OS model is a new preclinical model of tumors. The tumor tissue from a patient is xenografted into immunodeficient mice to generate tumor clones in the mice.^32^ This model preserves not only the molecular phenotype and genotype of the tumor itself but also the pathological differentiation and tumor heterogeneity.^32^ Therefore, the PDX model is a good way to address the issues of OS heterogeneity and pathological differentiation.^12^

In this study, we found that PLK2 could not only modulate the osteogenic differentiation and maturation of the conventional OS cell‐line Saos2 through enriched TAp73 but also affect PDX‐OS cell osteogenic differentiation and maturation, clone formation, and proliferation. In addition, PLK2 inhibition could also slow tumor growth and prolong the median survival of the PDX host. In conclusion, we found a new osteogenic differentiation mechanism, the TAp73/PLK2 axis, that coregulates the maturation of OS osteogenic differentiation, and suggest that PLK2 is a potential target for OS differentiation therapy.

## 2 MATERIALS AND METHODS

### 2.1 Chemicals, OS cell lines, cell culture, cell transfection, RT‐PCR, Western blotting (WB), and indirect immunofluorescence assays

The assays, cells, chemicals, and drugs used in this study are described in a previous article.^11^, ^46^ In brief, Saos2 cells, a conventional human OS cell line with a p53‐null mutation, were cultured in McCoy's 5A medium supplemented with 10% fetal bovine serum and 1% penicillin and streptomycin. The incubator was set at 37°C and 5% CO_2_, and the medium was changed as needed. Cells were inoculated and grown to 60%‐70% confluence before they were treated with the indicated molecules for 24 hours. The DNA‐damaging reagent CDDP was prepared in cell culture medium or PBS as needed. ELN582646, a PLK2 inhibitor, was diluted to 5 μg/mL in cell culture medium. Cells were transfected with Lipofectamine 2000 according to established protocols and verified by RT‐PCR or WB. For PCR, a HiScript II Q RT SuperMix kit for qPCR was used to detect first‐strand cDNA. SYBR Green was used to detect dsDNA products. mRNA products were standardized against the levels of the housekeeping gene β‐actin. The PCR primers for PLK2 and TAp73 were described in a previous article. The PCR primers for OCN were as follows: forward 5′‐CGCTACCTGTATCAATGGCTGG‐3′, and reverse 5′‐CTCCTGAAAGCCGATGTGGTCA‐3′; and forward 5′‐CGAGGTGATAGTGTGGTTTATGG‐3′; and reverse 5′‐GCACCATTCAACTCCTCGCTTTC‐3′. A CCK‐8 assay kit was used to measure cell proliferation. Indirect immunofluorescence experiments were performed as described previously.^46^ The following primary antibodies were used for WB: anti‐PLK2 (epr10070, ab154794, Abcam), anti‐TAp73 (38c674.2, ab79078, Abcam), anti‐OCN (MA1‐43028, 5021, Life Technologies Corporation), anti‐OPN (# 702184, 24H5L3, Life Technologies Corporation), anti‐Sox9 (# 702016, 7H13L8, Life Technologies Corporation), anti‐Collagen2 (# MA5‐12786, 2B1.5, Life Technologies Corporation), and anti‐β‐actin (a5441, Sigma‐Aldrich). The following primary antibodies were used in cell immunofluorescence assays: anti‐PLK2 (epr10070, ab154794, Abcam) and anti‐TAp73 (1E8, ab118985, Abcam). Secondary antibodies, including anti‐rabbit‐594 (zf‐0416, zsgbbio) and anti‐mouse‐488 (zf‐0412, zsgbbio), were selected based on the species of the primary antibody.

### 2.2 Patient specimens

From 2008 to 2019, 36 fresh HCOS specimens were collected from patients prior to chemotherapy at Shaoguan First People's Hospital, Zhujiang Hospital, Guangdong provincial people's Hospital, the Third Affiliated Hospital of Southern Medical University, Yuebei people's Hospital, Dongguan Eighth People's Hospital, and Guangdong People's Hospital. All experiments were approved by the ethics committee of Shaoguan First People's Hospital Affiliated to Southern Medical University, and all patients provided informed consent.

### 2.3 Acquisition of primary cells and the culture and passage of PDX‐OS cells

Isolation of primary PDX‐OS cells was performed according to protocols described in a previous article.^55^ In brief, three OS tissue samples approximately 0.1 cm^3^ in size were excised from PDX, and immediately placed in a sterile container filled with sterile saline on ice. After the samples were cleaned, they were transferred to a 15‐mL sterile centrifuge tube filled with 6 mL of culture medium and then sent to the laboratory for further processing. The OS tissues were then cut into 2‐mm^3^ pieces with sterile ophthalmic scissors, cleaned with sterile PBS, trimmed into fine tissue chips with sterile ophthalmic scissors, and rinsed with PBS many times until the liquid was clear, free of oil, and not turbid. The OS tissues were submerged in 3 mL of serum‐free medium containing 1 mg/mL collagen hydrolase and digested in a 37°C water bath for 30 minutes. After enzymolysis, 3 mL of culture medium containing 10% fetal bovine serum was added to neutralize the remaining collagen hydrolase. The samples were centrifuged at 350× *g* at room temperature for 5 minutes, and the supernatant was discarded; this process was repeated twice. The cell pellets obtained after centrifugation were resuspended in PBS, counted and seeded into T25 cell culture flasks. The culture medium was changed either every 2 to 3 days or when the color of the medium in the culture flask was profoundly different. Then, the cultures were expanded, passaged, and preserved. All experiments involving primary cells were conducted within the first 10 passages. PDX‐OS cells were cultured in DMEM (Invitrogen) supplemented with 10% fetal bovine serum and 1% penicillin and streptomycin. The incubator was set at 37°C and 5% CO_2_.

### 2.4 Alizarin red staining assay

Calcium deposition was detected with alizarin red dye at an absorbance of 570 nm, and mineralization was determined with an Alizarin Red Staining Kit (Catalog #: kga363‐1, Keygen Biotechnology Company) as previously described.^56^ In brief, cells were seeded in 6‐well plates at a density of 3 × 10^5^ cells/well. After 36 hours, when the cells reached 90% confluence, they were washed with PBS and fixed with 70% ethanol for 1 hour at room temperature. After another wash with PBS, a 1% alizarin red solution was incubated with the cells at 37°C for 1 hour and fixed. To precipitate the dye, the cells were incubated with 10% cetylpyridinium chloride for 30 minutes at room temperature. The extent of calcium deposition was determined by using a microplate spectrophotometer (BMG LabTech, Germany) to measure the optical density (OD) at 570 nm.

### 2.5 Clonogenic assay

Cells were plated at 1000 cells/well in 6‐well plates. Each cell line was plated in triplicate and incubated for 24 hours to allow the cells to attach to the dish. Then, the cells were treated with an siRNA or a plasmid. Empty vector was included as a negative control. To promote tumor cell growth, the culture medium was replaced with keratinocyte‐SFM (Gibco) containing EGF (10 ng/mL) and FGF (5 ng/mL) (StemCell). After 14 days, the cells were washed, fixed, and stained with 0.5% crystal violet according to the manufacturer's instructions. Colonies with ≥50 cells were counted in the wells.

### 2.6 PDX animal experiment

Female BALB/c nude mice aged 4‐6 weeks were obtained from the Laboratory Animal Center of Southern Medical University, China. All mice were raised in animal facilities approved by Southern Medical University and in accordance with the guidelines for the care and use of laboratory animals. The experimental steps were detailed previously.^45^ In brief, a 2‐mm^3^ PDX‐OS tissue specimen was inoculated into the right femurs of mice. When the xenograft tumor volume reached approximately 350 mm^3^, we began to treat the tumors (6 mice per group). Animals were intraperitoneally injected with CDDP (5 mg/kg, 0.9% isotonic saline solution), administered a PLK2 inhibitor via oral gavage (100 mg/kg), or treated with both compounds. All drugs were freshly prepared twice a week over a 28‐day period. The mice in the control group received only vehicle. Tumor volume (mm^3^) and weight were measured until the mice died or the experiment was terminated.

### 2.7 Hematoxylin and eosin (HE) staining and immunohistochemical (IHC) analysis

The details are described in a previous article.^57^ Pathological HE staining was carried out according to a standard procedure. In short, formalin‐fixed sections were dehydrated, stained with a hematoxylin solution for 5 minutes, soaked in 1% acid in ethanol (1% HCl in 70% ethanol) 5 times, rinsed in distilled water, stained with an eosin solution for 3 minutes, dehydrated with different concentrations of alcohol and washed with xylene. The slides were then examined under an Olympus bx53 fluorescence microscope (Tokyo, Japan). The extent of staining and staining intensity of TAp73 and PLK2 in OS tissues were measured by IHC analysis.

Methods and procedures are detailed in the literature.^57^ The staining method involving streptomycin avidin‐peroxidase was used. In short, paraffin sections were dewaxed and rehydrated according to a standard protocol. After antigen retrieval with heated citric acid buffer, the sections were naturally cooled to room temperature and then rinsed with PBS. Then, 3% hydrogen peroxide was added to block endogenous peroxidase activity (37°C, 10 minutes). The sections were rinsed with PBS, goat serum was added to block nonspecific binding (37°C, incubation for 40 minutes), and the blocking solution was removed. A mouse anti‐human TAp73 antibody and rabbit anti‐human PLK2 antibody were added at a dilution of 1:200 for both antibodies, and the sections were incubated at 4°C overnight, followed by rewarming at 37°C for 45 minutes. PBS washing was performed three times, and anti‐mouse or anti‐rabbit secondary antibody was added (37°C, incubation for 60 minutes). PBS washes were performed three times, followed by DAB visualization, hematoxylin staining, dehydration and clearing, and neutral gum sealing of the sections. Sections of positive specimens were used as a positive control, and sections treated with PBS buffer instead of a primary antibody served as a negative control. All reagents were obtained from Vector Laboratories. The rate of positive protein expression was evaluated according to the percentage of positive cells and the intensity of staining. The percentage of positive cells was scored as follows: 0, less than 10% positive cells; 1, 10%~25% positive cells; 2, 26%~50% positive cells; and 3, more than 50% positive cells. The staining intensity was scored as follows: 0, no staining; 1, light yellow; 2, yellow; and 3, dark yellow to brown. The total score was calculated by adding the positive cell percentage score and staining intensity score and was classified as follows: 0, negative (‐); 1‐2, weakly positive (+); 3‐4, positive (+ +); and 5‐6, strongly positive (+ + +). More broadly, (‐) and (+) were defined as low expression, whereas (+ +) and (+ + +) were defined as high expression.

### 2.8 Statistical analysis

Statistical analysis of the data was performed as described in a previous article.^46^ In brief, each assay was carried out three times. All data involving multiple groups were analyzed by ANOVA and Dunnett's test, and data from two groups were compared by a student's test or chi‐square test. *P* < .05 was considered statistically significant.

## 3 RESULTS

### 3.1 The heterogeneity of PLK2 and TAp73 expression in HCOS, which is related to the maldifferentiation of osteoblastic osteosarcoma and chondroblastic osteosarcoma, affects patient prognosis

**FIGURE 1:**
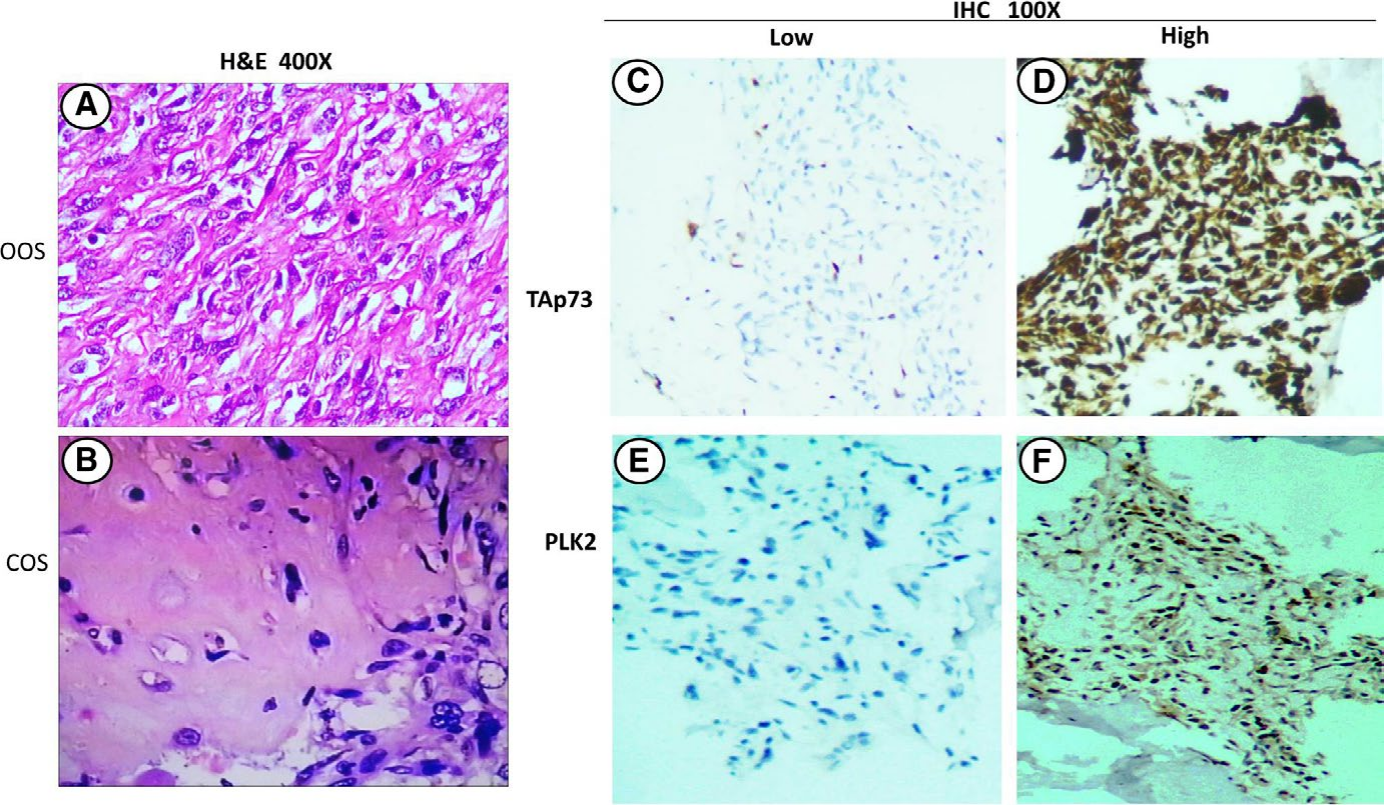
Hematoxylin and eosin (HE) and immunohistochemical (IHC) staining of human conventional osteosarcoma (HCOS). A, HE staining of osteoblastic osteosarcoma (OOS; 400×). B, HE staining of chondroblastic osteosarcoma (COS; 400×). C, Low expression of TAp73 (100×) as detected by IHC analysis. D, High expression of TAp73 (100×) in HCOS as detected by IHC analysis. E, IHC analysis showing that PLK2 was expressed at low levels (100×). F, High expression of PLK2 (100×) in HCOS as detected by IHC analysis

We used immunohistochemistry to assess TAp73 and PLK2 levels in 36 specimens of HCOS, including 23 samples of OOS and 13 samples of COS (Figure 1).

Regarding TAp73 or PLK2 expression alone (Table 1), the data showed that high expression of TAp73 occurred in 72.22% (26/36) of the HCOS specimens, and high PLK2 expression occurred in 41.67% (25/36). In OOS, the rates of high TAp73 and PLK2 expression were 82.61% (19/23) and 21.74% (5/23), respectively. Moreover, in COS, the rate of high TAp73 expression was 53.85% (7/13), while that for high PLK2 expression was 76.92% (10/13). There were statistically significant differences (*P* < .05).

**TABLE 1:** TAp73 and PLK2 expression in 36 cases of human conventional osteosarcoma

HCOS	Total (n)	TAp73		PLK2	
		Low	High	Low	High
OOS	23	4	19	18	5
COS	13	6	7	3	10
χ2		3.425		10.41	
*P*		.0321		.0013	

Abbreviations: COS, chondroblastic osteosarcoma; HCOS, human conventional osteosarcoma; OOS, osteoblastic osteosarcoma.

Combined analysis of TAp73 and PLK2 expression in HCOS (Table 2) showed that 13.89% (5/36) of the specimens exhibited simultaneous low expression of TAp73 and PLK2, whereas 44.44% (16/36) had high expression of TAp73 and low expression of PLK2. By contrast, 13.89% (5/36) of the specimens presented low expression of TAp73 and high expression of PLK2, whereas 27.78% (10/36) showed simultaneous high expression of TAp73 and PLK2. In OOS, the rate of simultaneous low expression of TAp73 and PLK2 was 8.7% (2/23), high expression of TAp73 and low expression of PLK2 was 69.57% (16/23), low expression of TAp73 and high expression of PLK2 was 8.7% (2/23), and simultaneous high expression of TAp73 and PLK2 was 13.04% (3/23). In COS, the distribution of these expression patters was 23.08% (3/13), 0, 23.08% (3/13), and 53.85% (7/13), respectively. There were statistically significant differences (*P* < .01).

**TABLE 2:** The heterogeneity of TAp73 and PLK2 expression in 36 cases of human conventional osteosarcoma

HCOS	Total (n)	Both TAp73 and PLK2 low	TAp73 high and PLK2 low	TAp73 low and PLK2 high	Both TAp73 and PLK2 high
OOS	23	2	16	2	3
COS	13	3	0	3	7
χ^2^		16.49			
*P*		0.0009			

Abbreviations: COS, chondroblastic osteosarcoma; HCOS, human conventional osteosarcoma; OOS, osteoblastic osteosarcoma.

In general, the above data indicated that the proportion of HCOS specimens with high TAp73 expression was greater than that with high PLK2 expression, especially in OOS. However, the proportion of COS specimens with high PLK2 expression was greater than that with high TAp73 expression. In addition, the most common expression pattern in HCOS was high expression of TAp73 and low expression of PLK2. There were no COS specimens with high expression of TAp73 and low expression of PLK2. However, in the COS specimens, simultaneous high expression of TAp73 and plk2 and low expression of TAp73 with high PLK2 expression were dominant, especially the former. The results suggest that the heterogeneity of TAp73 and PLK2 expression in OOS and COS contributes to the poor pathological differentiation.

**FIGURE 2:**
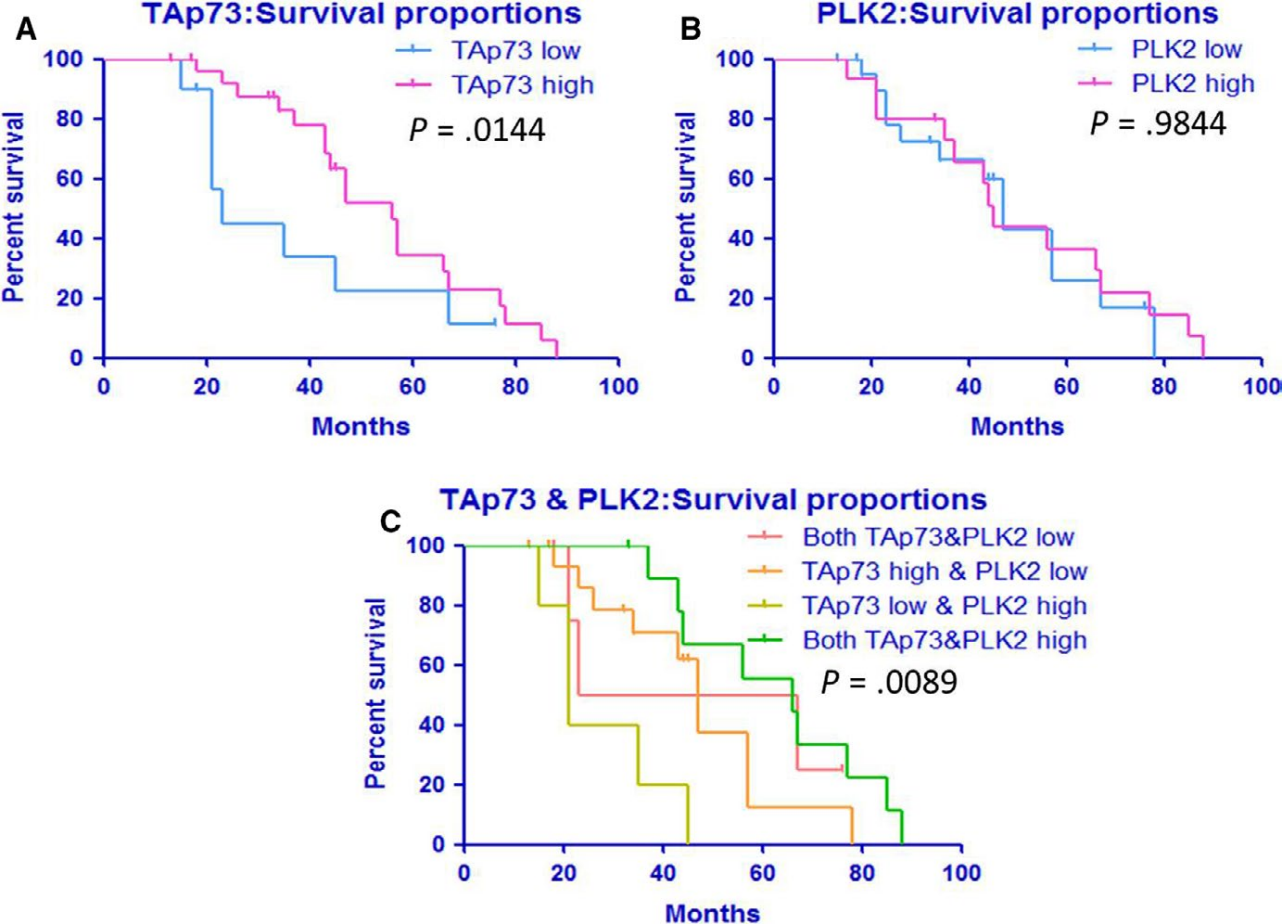
Heterogeneous expression of TAp73 and PLK2 is correlated with median survival in HCOS patients. A, Median survival was 56 months for patients with high TAp73 expression and 23 months for patients with low TAp73 expression (*P* < .05). B, Median survival was 45 months for patients with high PLK2 expression and 47 months for patients with low PLK2 expression (*P* > .05). C, Combined analysis of TAp73 and PLK2 showed that the median survival of patients with high expression of both TAp73 and PLK2 was 66 months, while that of patients with low expression of TAp73 but high expression of PLK2 was 21 months. In addition, patients with high expression of TAp73 but low expression of PLK2 had a median survival of 47 months. Patients with low expression of both TAp73 and PLK2 had a median survival of was 45 months (*P* < .01)

Kaplan‐Meier survival analysis (Figure 2A) showed that the overall survival of HCOS patients with high TAp73 expression (median survival: 56 months) was longer than that of patients with low TAp73 expression (median survival: 23 months), and the difference was statistically significant (*P* < .05). PLK2 expression had no significant effect on the overall survival of HCOS patients (Figure 2B) (median survival of patients with high expression or low expression of PLK2 was 45 months and 47 months, respectively). These data indicate that when PLK2 is highly expressed, the abundance of TAp73 expression has a significant effect on the median survival of patients (Figure 2C). Spearman correlation analysis showed that the survival of HCOS patients was positively correlated with TAp73 expression (r = 0.46, *P* = .018), and there was a positive correlation between TAp73 and OOS/COS (r = 0.5954, *P* < .01). By contrast, Spearman correlation analysis of PLK2 and OOS/COS showed that OOS/COS was negatively correlated with PLK2 expression (r = −0.4937, *P* < .01). The results showed that the expression level of TAp73 affected the median survival of HCOS patients and that the heterogeneity of TAp73 expression in relation to PLK2 expression might be correlated with osteogenic or chondrogenic differentiation of HCOS.

### 3.2 In Saos2 and PDX‐OS cells, the interaction between PLK2 and TAp73 at high abundance affects osteogenic differentiation and maturation

**FIGURE 3:**
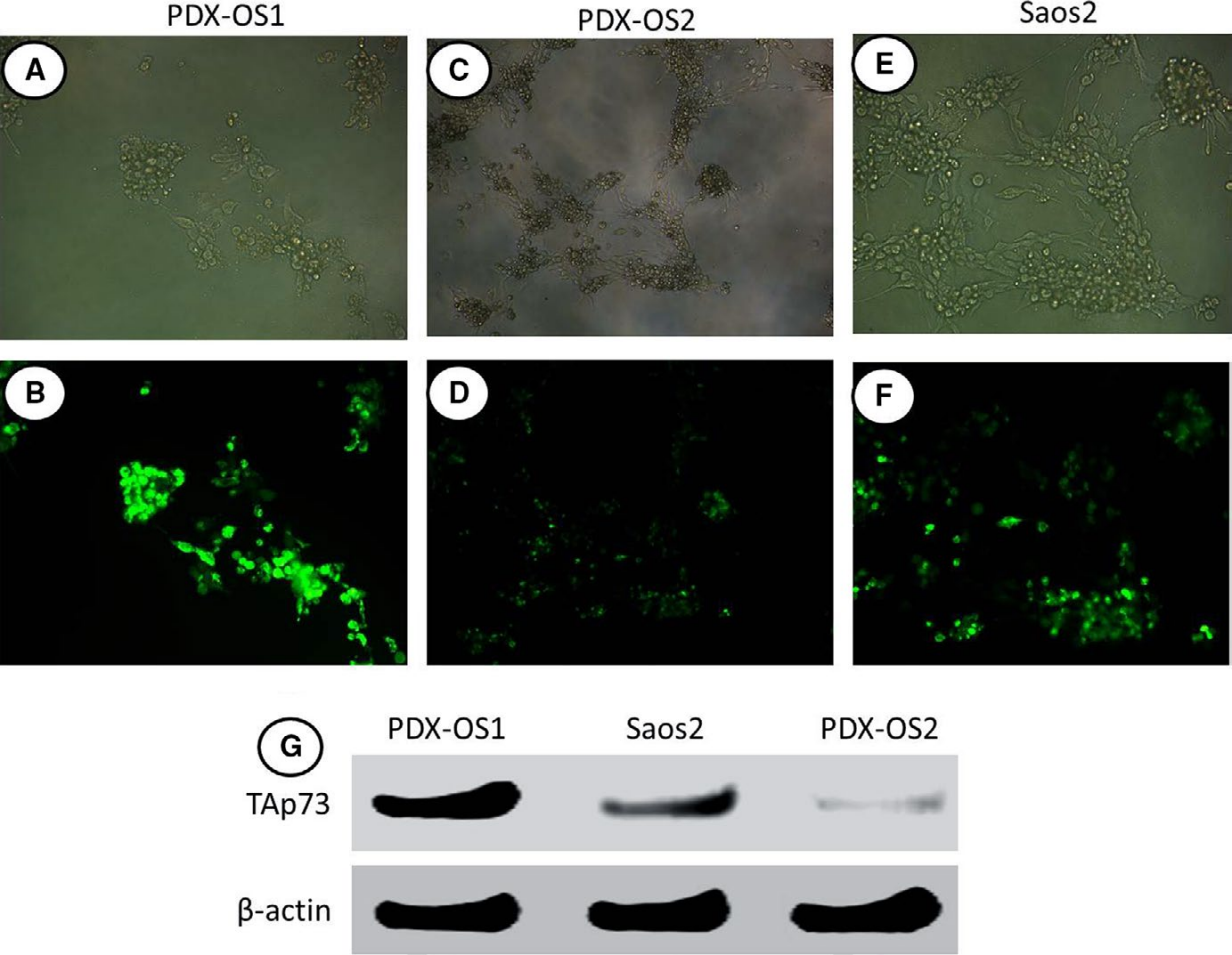
There were different levels of TAp73 expression among the conventional OS cell line Saos2 and two OS cell lines generated from patient‐derived xenograft (PDX) tissues under normal culture conditions. A and B, PDX‐OS1 cells showed a high abundance of TAp73 expression under normal culture conditions as observed by inverted‐phase microscopy (A) and TAp73‐specific immunofluorescence staining (green) (B). C and D, PDX‐OS2 cells showed a low abundance of TAp73 expression under normal culture conditions as observed by inverted‐phase microscopy (B) and TAp73‐specific fluorescence imaging (green) (C). E and F, Saos2 cells showed a moderate abundance of TAp73 expression as observed by inverted‐phase microscopy (E) and TAp73‐specific fluorescence imaging (green) (F). G, A total of 1 × 10^5^ cells were seeded in a cell culture dish and cultured for two days until they reached 80% confluence, at which point they were harvested and lysed. The isolated protein concentration was quantified, and electrophoresis was performed. The amount of protein in each well was 30 μg. After the proteins were transferred to a membrane, TAp73 immunoblotting was performed to assess the relative abundance of TAp73 in the three OS cell lines, consistent with the immunofluorescence assay

Previous studies have shown that PLK2 can phosphorylate TAp73 and affect its transcriptional activity.^46^ To observe whether the interaction between PLK2 and TAp73 affects the osteogenic differentiation and maturation of OS cells, we used the conventional p53‐null cell‐line Saos2 and the PDX‐OS1 and PDX‐OS2 cell lines derived from PDX tissues and measured TAp73 expression via immunofluorescence and WB assays. TAp73 expression in these three OS cell lines was heterogeneous (Figure 3). In the immunofluorescence cell assay, the TAp73 signal was strongest in PDX‐OS1 cells (Figure 3A,B), weakest in PDX‐OS2 cells (Figure 3C,D), and moderate in Saos2 cells, with a level between those of the two PDX‐OS cell lines (Figure 3E,F). The WB results verified that TAp73 expression in the three OS cell lines was different under basic culture conditions. PDX‐OS1 cells had a significantly higher abundance of TAp73 than Saos2 cells, whereas PDX‐OS2 cells had a lower abundance than Saos2 cells.

**FIGURE 4:**
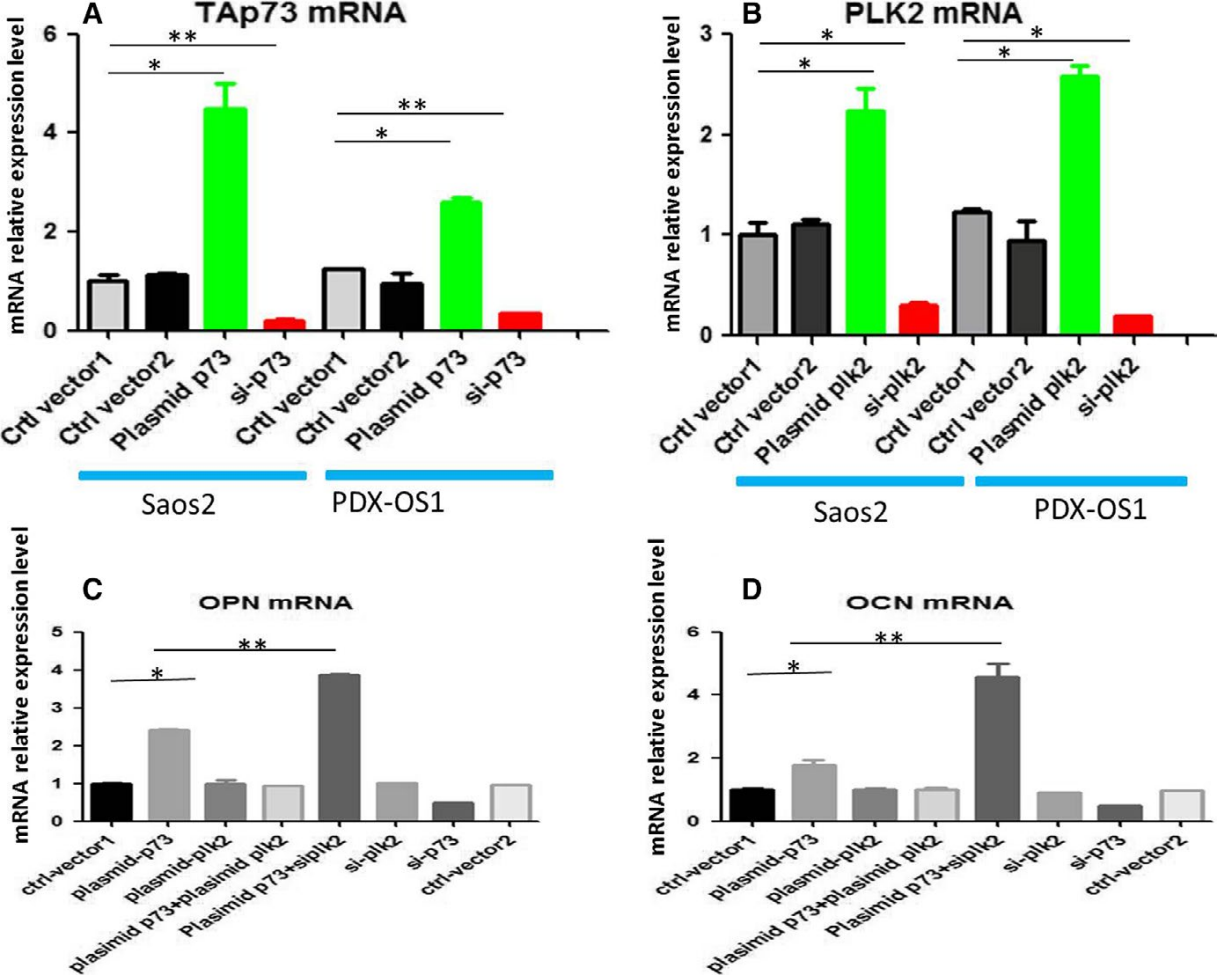
The mRNA expression levels of TAp73 and PLK2 affected OPN and OCN mRNA expression in Saos2 and PDX‐OS1 cells. A, In Saos2 and PDX‐OS1 cells, the mRNA expression of TAp73 was upregulated by plasmid‐p73 and downregulated by siRNA‐p73 (si‐p73). B, In Saos2 and PDX‐OS1 cells, the mRNA expression of PLK2 was upregulated by plasmid‐plk2 and downregulated by siRNA‐plk2 (si‐plk2). C, OPN mRNA expression increased when TAp73 mRNA expression was upregulated, while OPN mRNA expression decreased when TAp73 mRNA expression was downregulated by si‐p73 in treated Saos2 cells compared with control cells. OPN mRNA expression did not change when PLK2 mRNA expression alone was upregulated or downregulated. Therefore, TAp73 and PLK2 levels were simultaneously upregulated. OPN mRNA expression increased more significantly when TAp73 expression was upregulated and PLK2 expression was downregulated. D, Similar to the OPN mRNA results, OCN mRNA expression increased when TAp73 mRNA expression alone was upregulated and decreased when TAp73 mRNA expression alone was downregulated; OCN mRNA was not significantly affected by the downregulation or upregulation of PLK2 alone. Moreover, when TAp73 mRNA expression was upregulated in combination with PLK2 mRNA downregulation, OCN mRNA expression was more significantly increased (*P < .05; **P < .01)

To observe the effect of the PLK2‐Tap73 interaction on the osteogenic differentiation of OS cells, we downregulated and upregulated the expression of PLK2 and/or TAp73 by transfecting the corresponding siRNAs or plasmids, respectively, into Saos2 and PDX‐OS cells. RT‐PCR data showed that these siRNAs significantly downregulated the expression of their target molecules, whereas the plasmids upregulated the abundance of TAp73 (Figure 4A) and PLK2 (Figure 4B) mRNA in Saos2 cells and in PDX‐OS1 cells (Figure 4A,B). In addition, the RT‐PCR assay data showed that the levels of the osteogenic differentiation markers OPN (Figure 4C) and OCN (Figure 4D) were significantly increased in Saos2 cells accompanied by an increase in TAp73 abundance induced by the TAp73 overexpressing plasmid but not by the PLK2 overexpressing plasmid alone. The OPN and OCN mRNA levels were not increased with the simultaneous upregulation of both TAp73 and PLK2 expression in Saos2 cells. However, the increases in OPN and OCN expression were most significant when TAp73 expression was upregulated in combination with downregulation of PLK2 expression. Moreover, similar RT‐PCR results were shown in PDX‐OS1 cells. Interestingly, due to the high abundance of TAp73 under basal conditions in PDX‐OS1 cells, the mRNA expression of OPN and OCN increased when PLK2 was downregulated alone (data not shown).

**FIGURE 5:**
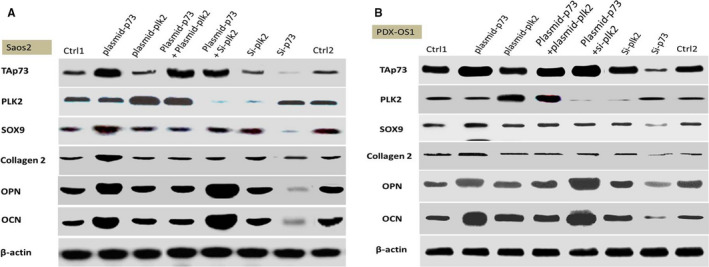
The abundance of TAp73 and PLK2 affected the protein expression of OPN, OCN, SOX9 and Collagen2 in Saos2 and PDX‐OS1 cells, as evaluated by WB. Cells were cultured to 60%‐70% confluence and then collected 24 hours after receiving the indicated treatment. Blank siRNA vector 1 (ctrl1) and blank plasmid vector 2 (ctrl2) were used as controls. A, In Saos2 cells, OPN, OCN, SOX9 and Collagen2 levels increased when TAp73 expression was increased compared with the control treatments, and the protein levels of decreased when TAp73 expression alone was decreased (*P* < .05). However, these changes did not occur if PLK2 expression alone was either increased or decreased. However, when TAp73 expression was increased and PLK2 expression was decreased, the increases in OPN and OCN expression were the most significant (*P* < .01), but the changes in SOX9 and Collagen2 expression were not significant. When the levels of both TAp73 and PLK2 were increased, OPN, OCN, SOX9 and Collagen2 levels did not increase. B, In PDX‐OS1 cells, similar to Saos2 cells, changes in TAp73 expression tended to be accompanied by changes in OPN, OCN, SOX9 and Collagen2 protein levels (*P* < .05). When TAp73 expression was increased in combination with a decrease in PLK2 expression, the increases in OPN and OCN expression were the most significant (*P* < .01), but changes in SOX9 and Collagen2 expression were not. By contrast, changes in OPN and OCN protein levels (*P* < .05) but not SOX9 or Collagen2 protein levels tended to be accompanied by changes in PLK2 alone

Next, WB was carried out (Figure 5), and the data showed that in Saos2 cells (Figure 5A), overexpression of TAp73 protein alone increased the protein levels of OPN, OCN, and the chondrogenic differentiation markers SOX9 and Collagen2, but overexpression of PLK2 alone did not. Similar to the RT‐PCR assay, WB showed that simultaneous overexpression of TAp73 and PLK2 did not increase the protein abundance of the OPN, OCN, SOX9, and Collagen2. When TAp73 overexpression was combined with PLK2 inhibition, the increases in the abundance of OPN and OCN peaked, but the SOX9 and Collagen2 protein levels did not increase. The abundance of OPN, OCN, SOX9, and Collagen2 decreased when only TAp73 was inhibited, and inhibition of PLK2 alone did not affect the abundance of the above protein markers.

PDX‐OS1 cells (Figure 5B) showed changes similar to those observed in Saos2 cells. The change in the abundance of the TAp73 protein was accompanied by changes in the protein levels of OPN, OCN, Sox9, and collagen 2. The main difference was that, as TAp73 is highly abundant in PDX‐OS1 cells under basal conditions, changes in the abundance of OPN and OCN could be observed by inhibiting PLK2 alone.

The results of the above molecular experiments indicate that the abundance of TAp73 has an effect on the expression of osteogenic and chondrogenic differentiation markers. In addition, the basal abundance of TAp73 was low in Saos2 cells, and targeting only PLK2 did not produce effects. However, in PDX‐OS1 cells (which have high basal TAp73 expression), PLK2 inhibition significantly increased the expression of osteogenic differentiation markers but not of chondrogenic differentiation markers.

**FIGURE 6:**
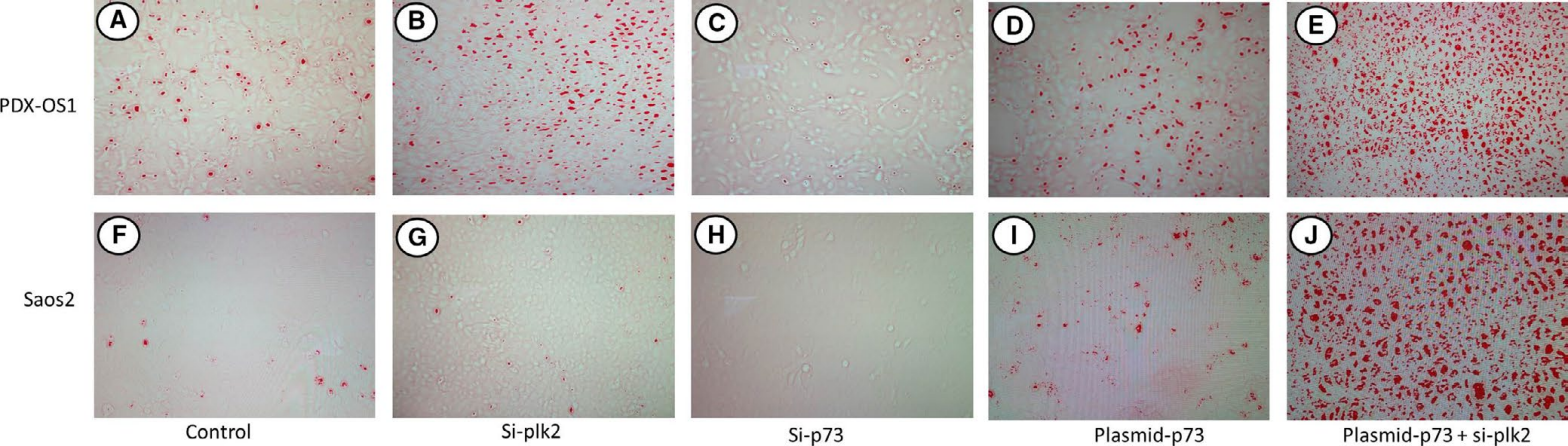
PLK2 inhibition promoted the abundance of TAp73 to regulate ossification maturation in OS cells. OS cells were cultured to 90% confluence, and calcification deposition staining was carried out. A blank vector was used as the control. A‐E, In PDX‐OS1 cells, compared with treatment with the corresponding control (A), PLK2 downregulation alone by si‐plk2 (B) or TAp73 upregulation alone by plasmid‐p73 (C) decreased the red calcium deposition signal (*P* < .05), while TAp73 downregulation alone by si‐p73 (D) significantly increased the red calcium deposition signal (*P* < .05). Moreover, TAp73 upregulation combined with PLK2 downregulation (E) resulted in the most significant increase in the red calcium deposition signal compared to the corresponding control (*P* < .01). F‐J, In Saos2 cells, PLK2 downregulated alone by si‐plk2 (G) did not affect the red calcium deposition signal compared with treatment with the corresponding control (F). TAp73 downregulation alone by si‐p73 (H) significantly decreased the red calcium deposition signal (*P* < .05), while TAp73 upregulation alone by plasmid p73 (I) significantly increased the red calcium deposition signal (*P* < .05). Consistently, TAp73 upregulation combined with PLK2 inhibition (J) resulted in the most significant increase in the red calcium deposition signal (*P* < .01)

Mineral deposition further indicates the maturation of cells undergoing osteogenic differentiation, namely, ossification. We observed the effect of PLK2 interacting with TAp73 on the ossification of OS cells with an alizarin red calcium deposition staining assay (Figure 6). The results showed that in PDX1‐OS cells with a high abundance of TAp73 under basal conditions (Figure 6A‐E), the group with only siRNA‐mediated PLK2 inhibition showed a significantly increased cell calcium deposition staining signal (Figure 6B) compared with that of the control group (Figure 6A). When only an siRNA targeting TAp73 was used, the calcium staining signal was decreased (Figure 6C). By contrast, when TAp73 was overexpressed with a plasmid, the calcium deposition staining signal was also increased significantly (Figure 6D). Moreover, TAp73 overexpression combined with PLK2 inhibition resulted in the most significant increase in the calcium deposition staining signal (Figure 6E). Similarly, in Saos2 cells (Figure 6F‐J), the inhibition or overexpression of TAp73 affected the calcium deposition signal. However, due to the low abundance of TAp73 in Saos2 cells under basal conditions, PLK2 inhibition alone did not significantly increase the calcium deposition signal. Therefore, the above results indicated that the interaction between PLK2 and TAp73 affects osteogenic differentiation and maturation.

### 3.3 The interaction between PLK2 and TAp73 affects the clonogenic and proliferative abilities of PDX‐OS cells

**FIGURE 7:**
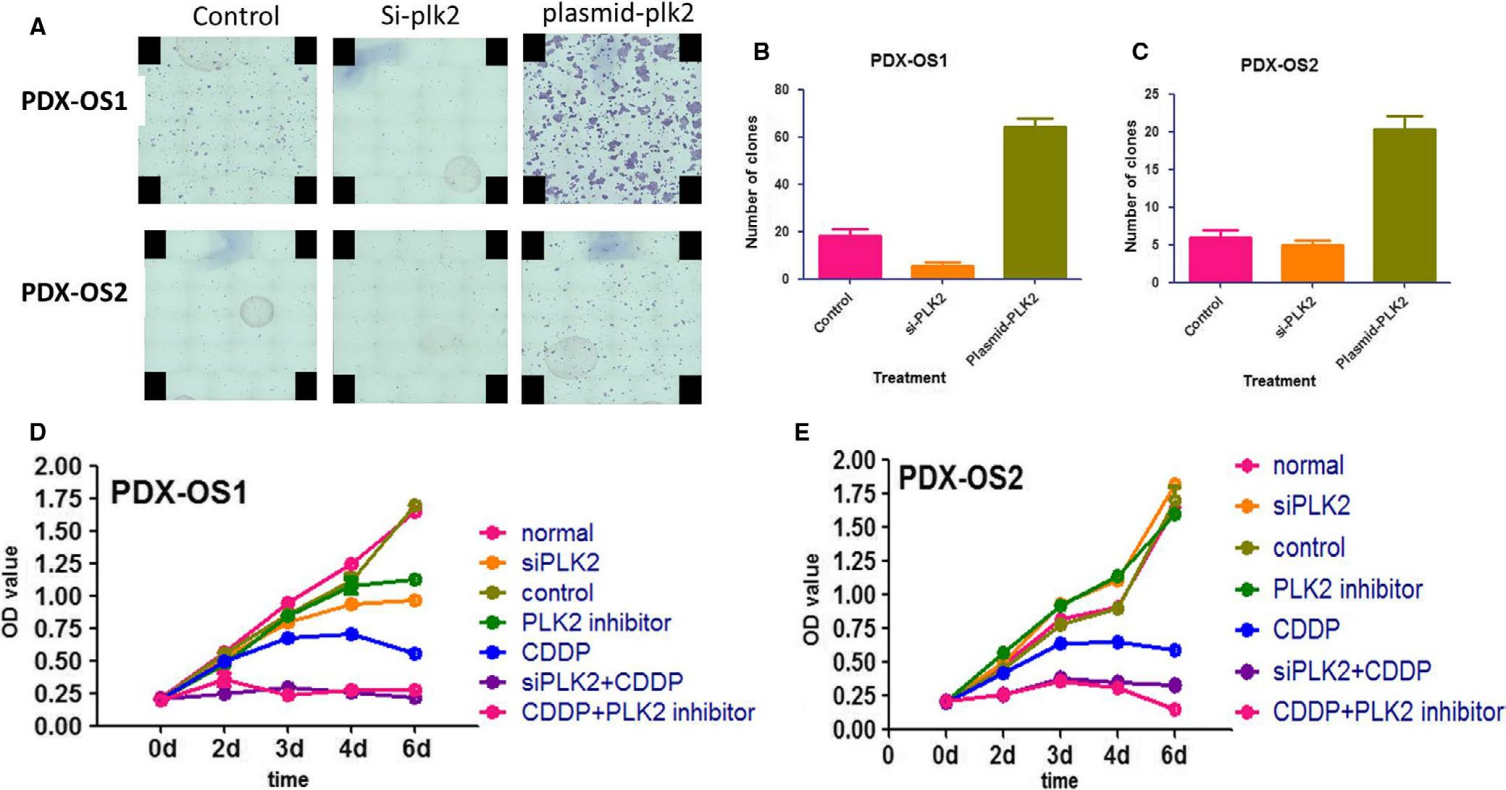
PLK2 regulated colony formation and sensitized PDX‐OS cells to CDDP in a proliferation assay. A and B, In PDX‐OS1 cells, compared with treatment with the corresponding control, PLK2 downregulation by si‐plk2 inhibited the formation of cell colonies (*P* < .01). By contrast, when PLK2 expression was upregulated by plasmid‐plk2, the formation of cell colonies was significantly increased (*P* < .01). A and C, Similar to PDX‐OS1 cell colony formation, PDX‐OX2 cell colony formation was affected by PLK2 downregulation or upregulation. The difference was statistically significant (*P* < .05). D, In a PDX‐OS1 cell proliferation assay, compared with the blank vector (control) and DMSO (normal) treatment groups, the siRNA2‐treated group (knocked down PLK2 expression) and PLK2 inhibitor‐treated group showed decreased OD values for cell proliferation in the later phase. However, CDDP treatment alone significantly reduced the OD value related to cell proliferation (*P* < .05), while CDDP combined with either PLK2‐specific siRNA or PLK2 inhibitor treatment reduced the OD value more significantly (*P* < .01). E, In PDX‐OS2 cells, compared with the control and normal groups, the PLK2‐specific siRNA group and PLK2 inhibitor‐treated group had no significant decreases in cell OD values. However, in the CDDP treatment group, the OD value was decreased significantly (*P* < .01), while in the groups treated with CDDP in combination with either PLK2‐specific siRNA or PLK2 inhibitor treatment, the OD values were decreased more significantly (*P* < .01)

Previous studies have shown that PLK2 inhibited the anti‐OS function of TAp73 at high abundance via phosphorylation and that inhibiting PLK2 promoted CDDP‐induced apoptosis of conventional OS cells.^46^ To observe whether there are similar effects on PDX‐OS1 and PDX‐OS2 cells, colony formation and proliferation assays were carried out (Figure 7). The OS cell clonogenic assay data showed that in PDX‐OS1 cells with high basal expression of TAp73 (Figure 7A,B), PLK2 inhibition significantly inhibited cell clonogenesis, while PLK2 overexpression enhanced cell clonogenesis. In PDX‐OS2 cells with a low basal abundance of TAp73, the results were basically consistent (Figure 7A,C).

On the other hand, compared with control treatment, CDDP significantly inhibited the proliferation of both PDX‐OS1 and PDX‐OS2 cells (Figure 7D,E). In PDX‐OS1 cells with high basal expression of TAp73 (Figure 7D), PLK2 inhibition alone prevented late OS cell proliferation, and CDDP combined with inhibition of PLK2 by either siRNA or a PLK2 inhibitor significantly inhibited tumor cell proliferation in both OS cell lines. However, in PDX‐OS2 cells with low basal expression of TAp73 (Figure 7E), inhibition of PLK2 alone had no significant effect on cell proliferation. The above assays indicate that PLK2 inhibition can enhance the anti‐OS effect of CDDP independent of the basal levels of TAp73 expression.

### 3.4 PLK2 inhibition combined with CDDP inhibits tumor growth and prolongs the median survival of animal hosts

**FIGURE 8:**
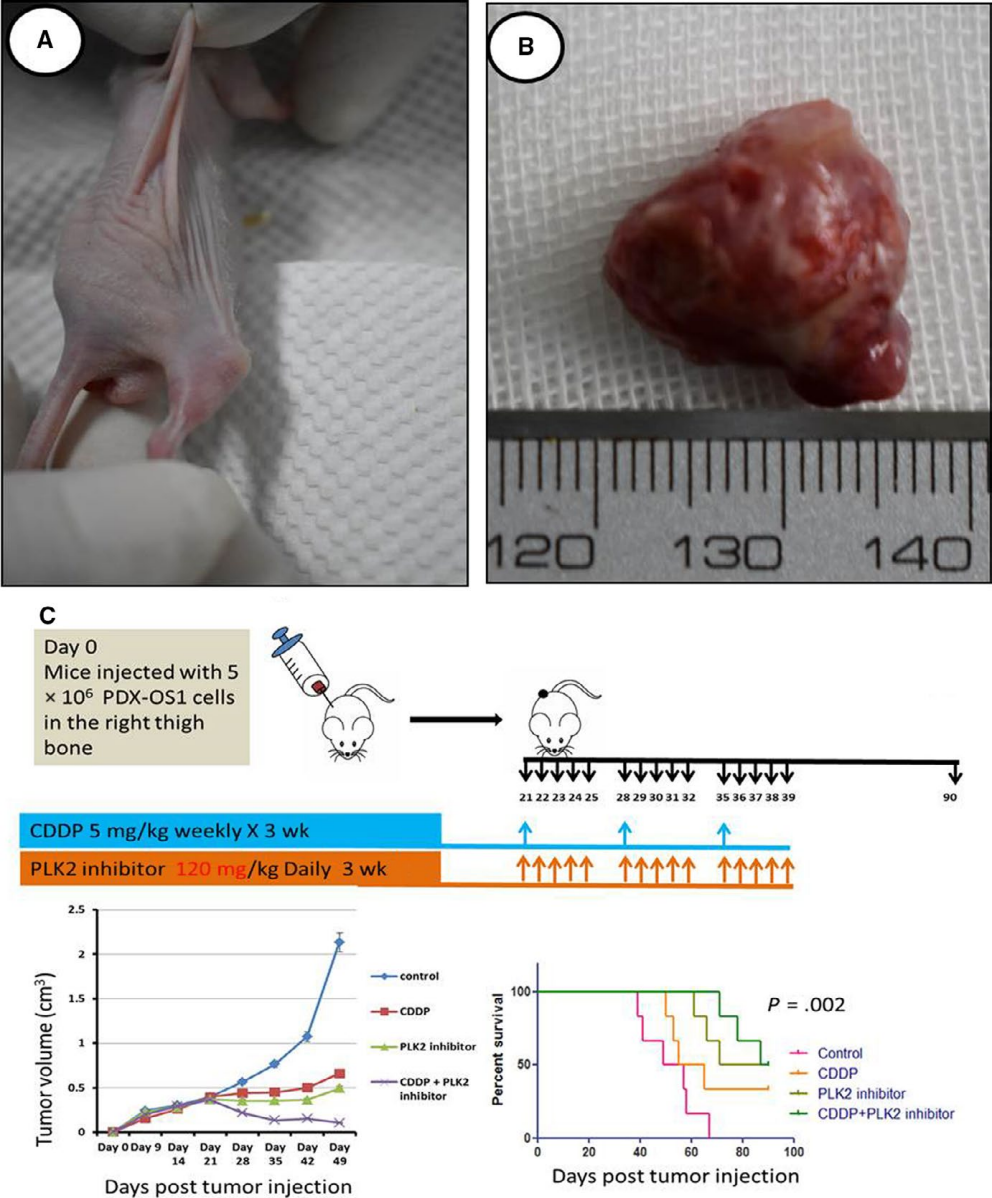
PLK2‐targeted therapy can promote CDDP‐mediated inhibition of OS growth in vivo and extend the median survival of hosts. A, An in situ PDX model with high TAp73 expression was established. B, Gross tumor specimens were obtained from the PDX model. C, When the tumor volume reached approximately 350 mm^3^, twenty‐four nude mice with PDX‐OS tumors were divided into 4 groups (6 mice per group). Compared with the control group, the CDDP alone and PLK2 inhibitor alone groups exhibited significantly reduced tumor growth (*P* < .01), while the CDDP combined with PLK2 inhibitor treatment group showed the most significant inhibition of tumor growth (*P* < .01). Survival analysis showed that the median survival of the CDDP treatment group was 60 days, that of the PLK2 inhibitor treatment group was 80.5 days and that of the combined treatment group was 88.5 days. The differences were statistically significant (*P* < .01)

CDDP, a chemotherapeutic drug that induces DNA damage, mediates its antitumor function through the p53/TAp73 family. To observe the effects of PLK2 alone and PLK2 combined with CDDP treatment in vivo, we constructed a nude mouse model of in situ PDX‐OS (Figure 8A,B) with OS patient tissues that had high basal expression of TAp73 and divided the mice into four treatment groups: control group, CDDP treatment group, PLK2 inhibitor treatment group, and CDDP combined with PLK2 inhibitor treatment group (6 mice per group) (Figure 8C). The data showed that in the control group, the tumor tissue grew rapidly, whereas in the CDDP treatment, PLK2 inhibitor treatment, and the combination treatment groups, tumor growth was significantly inhibited (*P* < .05). The inhibitory effect was most significant in the CDDP combined with PLK2 inhibitor group (*P* < .01); compared with the median survival of the control group (median survival time: 53 days), the survival of the CDDP (60 days), PLK2 inhibitor treatment group (80.5 days), and the combined treatment group (88.5 days) were longer. Therefore, the results of the animal experiments indicated that in OS with high basal expression of TAp73, CDDP had a significant anti‐OS effect, and inhibition of PLK2 could sensitize OS to CDDP and prolong median survival.

## 4 DISCUSSION

In this study, we analyzed the immunohistochemistry data of the tumor suppressor gene TAp73 and the tumor‐promoting gene PLK2 in clinical specimens and found that there is heterogeneity in the expression of TAp73 and PLK2 in OOS and COS cells, which affects the prognosis of patients. Subsequently, we found that the abundance of TAp73 expression affected the expression of the osteogenic differentiation markers OPN and OCN, as well as the chondrogenic differentiation markers Sox9 and collagen2. PLK2 only affected the expression of OPN and OCN via the abundance of TAp73, which further conditioned the maturation of osteogenic differentiation of OS cells, as indicated by the bone mineral deposition assay. These changes further affect the proliferation and clonal ability of OS cells. In the animal experiment of PDX‐OS cells with high expression of TAp73, inhibiting PLK2 can significantly inhibit the growth of OS tumors, prolong the survival of the host, and significantly enhance the antitumor effect of CDDP. Based on these data, we hypothesized that PLK2 is a potential target for differentiation therapy of OS.

It is known that TAp73, like its family member p53, is a natural antitumor gene. Although TAp73 is rarely mutated,^34^, ^35^, ^36^ there is a significant difference in the expression of TAp73 among tumor cells.^42^, ^43^, ^44^, ^46^ In this study, the immunohistochemistry data of 36 HCOS specimens showed that 72.22% exhibited high expression of TAp73. This may be closely related to more than 70% of tumor cells with p53 mutations and deletions.^33^ It should be noted that in the two pathological maldifferentiation subtypes of HCOS, the percentage of OOS specimens with high TAp73 expression is significantly greater than that in the COS specimens. On the other hand, previous studies indicated that PLK2 is a tumor‐promoting factor ^58^, ^59^, ^60^ that can phosphorylate and inhibit the anti‐OS function of TAp73.^46^ High expression of PLK2 in tumor cells can promote tumor progression and drug resistance and affect the prognosis of patients.^16^, ^61^ In this study, the immunohistochemistry data of PLK2 in HCOS showed that, compared with COS, OOS has a higher percentage of specimens with elevated TAp73 expression, while PLK2 expression exhibits the opposite pattern. Correlation analysis showed that high TAp73 expression and low PLK2 expression were positively correlated with OOS and negatively correlated with COS. In addition, similar to patients with other tumors,^17^, ^62^ HCOS patients with high TAp73 expression had a longer median survival than did patients with low expression of TAp73. However, other tumor studies have shown that the expression of PLK2, as a tumor promoter, has a controversial effect on the survival and prognosis of patients.^16^, ^60^, ^61^, ^63^, ^64^ Our analysis of the immunohistochemistry data showed that the expression of PLK2 had no significant effect on the median survival of patients. However, the combined analysis showed that the median survival of patients with high expression of both PLK2 and TAp73 was better than that of patients with low expression of both proteins, and both of these scenarios were better than that of patients with high expression of PLK2 and low expression of TAp73. The survival of patients with high expression of TAp73 and low expression of PLK2 was between the two extremes stated above. Therefore, the immunohistochemical data indicate that the effect of PLK2 on the survival and prognosis of HCOS patients may depend on the abundance of TAp73, and there are significant differences in the expression of TAp73 and PK2 in two subtypes of HCOS, namely, OOS and COS. However, whether this is related to the poor pathological differentiation of HCOS has not been reported.

Similar to other poorly differentiated tumor cells,^37^, ^65^, ^66^ OOS and COS are malignant tumors with dysregulated differentiation and abnormal proliferation.^67^, ^68^, ^69^, ^70^ Induction of differentiation and maturation would reduce the degree of malignancy, enhance the therapeutic effect, and improve the prognosis of patients.^21^, ^22^, ^23^, ^24^, ^25^ Previous studies ^71^ have shown that OPN and OCN, which are markers of osteogenic differentiation, are expressed at low levels in OS cells but at high levels in osteoblasts that have completed osteogenic differentiation and matured to ossification.^72^, ^73^, ^74^ Consistent with previous studies,^72^, ^73^, ^74^ this study found that the basal levels of osteogenic or chondrogenic differentiation markers were low in the conventional OS cell‐line Saos2 and in PDX‐OS cells. However, the abundance of TAp73 was different among these OS cells. This finding is consistent with that of other tumor cells.^42^, ^43^, ^44^ It is known that the antitumor gene TP53 is closely related to the formation of OS,^33^, ^75^ and its family gene TAp73 not only has partial antitumor functions ^34^, ^35^, ^36^ but is also closely related to the development and maturation of bone.^38^, ^39^, ^40^ It has been reported that TAp73 is highly expressed and loses its antitumor function,^42^, ^43^, ^44^ which may be related to the other roles of TAp73, such as regulating the differentiation of tumor stem cells and the osteogenic phenotype of stem cells.^47^, ^71^ In addition, it has also been reported that PLK2 affects individual bone development ^45^ and cartilage formation.^76^ Therefore, PLK2 and TAp73 may be related to the dysregulated differentiation of OS. Our results from the RT‐PCR and WB assays showed that TAp73 overexpression was accompanied by increases in OPN and OCN mRNA and protein levels, and these increases were more significant when TAp73 upregulation was simultaneously combined with PLK2 inhibition. These experiments confirmed that TAp73 can regulate the differentiation of OS cells. Moreover, our new finding is that PLK2 promotes osteogenic differentiation of OS when there is a high abundance of TAp73. In addition, the alizarin red staining assay showed that the expression of TAp73 in Saos2 and PDX‐OS1 cells was consistent with the level of calcium deposition and that the inhibition of PLK2 in OS cells with a high abundance of TAp73 enhanced calcium deposition. Thus, these data indicate that PLK2 modulates the maturation of osteogenic differentiation of OS cells with enriched TAp73. This is the most important point because the irreversible osteogenic differentiation and maturation of OS cells reduces the malignancy of the tumor.^20^, ^23^, ^24^, ^25^ Therefore, in combination with the above studies, PLK2 modulates OS osteogenic differentiation and maturation based on the high abundance of TAp73.

Many studies have shown that in multiple tumors, such as breast cancer, head and neck squamous cell carcinoma, gastric cancer, and leukemia, tumor cell proliferation is inhibited by inducing tumor cell differentiation and maturation to improve survival and prognosis.^21^, ^22^, ^77^, ^78^, ^79^ Similarly, OS cells show reduced proliferation and increased sensitivity to chemotherapy drugs when osteogenic differentiation is induced.^68^, ^80^ Previous studies ^46^ verified that PLK2 could inhibit the functional activity of TAp73 against OS, reduce apoptosis in tumor cells, and maintain the proliferation of OS cells. Consistent with the findings of previous studies, the results of this work showed that PLK2 promoted clone formation in PDX‐OS cells, indicating that PLK2 has a protective effect on tumor cells. PDX‐OS1 and PDX‐OS2 cell proliferation experiments showed that the effect of PLK2 on PDX‐OS cell proliferation was related to the abundance of TAp73. CDDP is a tumor DNA‐damaging agent that can activate p53/p73 signaling pathways to inhibit the proliferation of OS cells,^11^, ^46^ and improve the expression of endogenous TAp73. PLK2 inhibition increases the functional activity of TAp73.^46^ Therefore, inhibition of PLK2 not only affects the cloning ability of OS cells but also promotes the osteogenic differentiation and maturation of OS cells by activating TAp73 which, when highly abundant, inhibits the proliferation and promotes the apoptosis of OS cells.

Although it is worth noting that large‐scale studies show extreme heterogeneity in TAp73 expression among OS patients, many studies have shown that the PDX model has a strong predictive value of tumor responses to drugs.^75^, ^81^ Some studies have shown that the genomic changes between a primary OS tumor and the corresponding PDX tumors are relatively stable, while the genomic changes between a primary OS tumor and the corresponding established PDX tumors are very small.^48^ In addition, genomic changes have also been found to be highly consistent through multiple generations of PDX specimens and their derived cell lines.^47^ Many results indicate that PDX models can be used as a reliable preclinical tool to evaluate specific treatments for OS patients.^75^, ^81^, ^82^, ^83^, ^84^ Our studies in PDX‐OS1 and PDX‐OS2 cells showed that the results from these cell lines were consistent with those of the conventional OS cell‐line Saos2, indicating that PLK2 is a therapeutic target in OS cells with high expression of TAp73. Moreover, we established a PDX model with a high basal abundance of TAp73 in nude mice to further verify the role of PLK2‐targeted therapy. In agreement with a previous study,^47^, ^75^ our study also showed that the experimental results of PDX tumors and their derived cells were consistent: in the PDX model with high expression of TAp73, the effect of inhibiting PLK2 was the same as the effect of CDDP, which was the inhibition of tumor growth and prolonged median survival of the hosts. CDDP combined with PLK2 inhibition was the most effective treatment in this model.

In summary, this study shows that the differences in the expression of TAp73 and PLK2 in two subtypes of HCOS with different pathological maldifferentiation activities, OOS and COS, is related to the survival and prognosis of patients; PLK2 regulates the osteogenic differentiation and maturation of OS cells based on a high abundance TAp73, thus affecting the cloning and proliferation of tumor cells and reducing the malignancy of tumors. In addition, PLK2 is a potential therapeutic target as shown in the PDX model; PLK2 inhibition can sensitize CDDP to OS and prolong the median survival of the host. Thus, PLK2 is an ideal target for OS differentiation therapy.

## CONFLICTS OF INTEREST

All the authors declare that they have no conflicts of interest.

## Data Availability

The data that support the findings of this study are available on request from the corresponding author. The data are not publicly available due to ethical restrictions or privacy.
